# Cognitive Biases in Blood-Injection-Injury Phobia: A Review

**DOI:** 10.3389/fpsyt.2021.678891

**Published:** 2021-07-13

**Authors:** Elinor Abado, Tatjana Aue, Hadas Okon-Singer

**Affiliations:** ^1^School of Psychological Sciences, University of Haifa, Haifa, Israel; ^2^The Integrated Brain and Behavior Research Center, University of Haifa, Haifa, Israel; ^3^Department of Psychology, University of Bern, Bern, Switzerland

**Keywords:** cognition, bias, review, anxiety disorders, specific phobia, blood injection injury phobia

## Abstract

Blood-injection-injury (BII) phobia can lead to avoidance of crucial medical procedures and to detrimental health consequences, even among health workers. Yet unlike other specific phobias, BII phobia has been understudied. Specifically, while cognitive biases have been extensively investigated in other anxiety disorders, little is known about the same biases in BII phobia. The current article reviews cognitive biases in BII phobia and suggest future directions for further study and treatment. The reviewed biases include attention, expectancy, memory, perception, and interpretation biases. The investigation of these biases is highly relevant, as cognitive biases have been found to interact with anxiety symptoms. Results showed that attention, expectancy, and memory biases are involved in BII phobia, while no studies were found on interpretation nor perception biases. Mixed results were found for attention bias, as different studies found different components of attention bias, while others found no attention bias at all. Similarly, some studies found a-priori/a-posteriori expectancy biases, while other studies found only one type of bias. A better understanding of the cognitive particularities of BII phobia may lead to better treatments and ultimately reduce avoidance of needles and blood-related situations, thereby enabling individuals with BII phobia to undergo potentially life-saving medical procedures.

## Introduction

Being afraid of blood, injections, and injuries seems intuitive. As in the case of spiders, snakes, heights, and closed spaces, blood-related stimuli are generally aversive and therefore potentially phobia-inducing. Yet being extremely fearful of or disgusted by blood-related stimuli is considered a mental disorder known as *blood-injection-injury (BII) phobia* (1). Studies have found that 4% of the US population have BII phobia (2) and that 20–50% of adolescents and 20–30% of young adults are afraid of needles [for a review and meta-analysis, see (3)]. Most people who are afraid of needles experience needle-related bodily symptoms, such as dry mouth, shortness of breath, sweat, nausea, and feeling faint or dizzy (4). Fear of BII-related stimuli and events can lead to dire health consequences such as avoidance of vital vaccines, even among healthcare workers (3), in addition to avoidance of blood tests, pain relief measures, and blood donation (4).

Anxiety disorders in general and specific phobias in particular have been studied extensively, especially their cognitive aspects. Nevertheless, BII phobia remains understudied. Indeed, BII phobia is often referred to as “the neglected one” (5) or “a neglected diagnosis” (6). In their review of the psychophysiology of BII phobia, Ritz et al. (7) included a subsection titled “cognitions—the neglected factor in BII phobia treatment research” (p. 64), further emphasizing the need for cognitive research on this disorder. This need is also reflected in the fact that most of the studies cited in this article are not recent, as BII phobia—or at least its cognitive aspects—has not been studied often. Thus, little is known about cognitive and emotional particularities in the processing of fear-evoking stimuli in BII, as also noted by Cisler et al. (8) in their review on this disorder.

BII phobia is often compared with other specific phobias, such as spider and snake phobias. Such comparisons between BII phobia and animal phobias, which have been investigated intensively, can help us understand whether BII phobia is indeed unique and qualitatively different from these other disorders, as first suggested by Sawchuk et al. (9). For instance, Tolin et al. (10) asked participants with BII phobia or spider phobia to rate various phobia-related pictures on fear and disgust scales. While participants with BII phobia exhibited strong feelings of disgust toward their phobia-relevant pictures, participants with spider phobia exhibited a mixture of both emotions, with fear being the more dominant emotion [see also (11), for similar findings in a non-clinical population]. Sawchuk et al. (12) reported similar results using discriminant function analyses, suggesting that these phobias are indeed qualitatively different. Nonetheless, both groups exhibited elevated disgust sensitivity compared to control participants.

Using simultaneous EEG and ECG-fMRI, Michałowski et al. (13) compared between three groups of participants: with spider fear, with social fear, and with BII fear. All fear groups exhibited increased brain responses toward fear-relevant stimuli compared with control participants. Additionally, compared to the control group, the three fear groups exhibited higher LPP amplitudes toward fear-relevant cues, as well as an overall greater P1 hypervigilance effect. Lastly, the fear groups differed in their cardiac responses toward fear-relevant stimuli. These results suggest differential engagement of cognitive evaluation and down-regulation strategies among the three fear groups.

Finally, using functional magnetic resonance imaging (fMRI), Caseras et al. (14) found distinct neurobiological substrates when comparing BII phobia with spider phobia: When viewing spider-related stimuli, participants with spider phobia exhibited increased activation in the dorsal anterior cingulate cortex and the anterior insula, compared to participants with BII phobia and healthy controls. In addition, when viewing images of blood/injection injuries, participants with BII phobia exhibited increased activation in the thalamus and visual/attention areas, compared with participants with spider phobia and controls. These findings suggest that different neurocognitive mechanisms underlie these distinct phobias, in turn suggesting that their respective treatments should differ.

The physiological aspects of BII phobia are debated. For instance, some studies suggest that BII phobia is characterized by biphasic cardiac activation manifested in increased heart rate (HR) and blood pressure (BP) upon exposure to the fear-evoking stimulus (i.e., sympathetic activation), followed by a sharp drop in both measures (i.e., parasympathetic activation), presumably leading to fainting, a response found only in BII [e.g., (15)]. Nonetheless, only a subgroup of individuals with BII fear/phobia faint, as studies show that 20.5% of participants with fear of needles reported fainting upon encountering a needle (4) and 56% of participants with BII phobia reported fainting in the past (5). Importantly, the empirical basis for the biphasic response has been criticized [for a review, see (7); see also (16), for a psychophysiological evaluation of fainting]. For instance, the study by Santini et al. (17) casts doubt on the role of the parasympathetic system in fainting.

In spite of a number of studies showing that disgust is relevant in BII phobia, the evidence is mixed and far from unanimous. For instance, in a review focusing on disgust and fear in different disorders (spider phobia, BII phobia, and obsessive compulsive disorder), Cisler et al. (18) concluded that both fear and disgust characterize the aforementioned disorders, but the magnitude of these emotions may depend on the response domain measured [i.e., facial expressions, HR, neural substrate, cognitive processes; see also (19), for a review on emotions and the role of disgust in BII phobia; for more on the role of disgust in various anxiety disorders and in healthy populations, see (11, 20)]. On the other hand, Vossbeck-Elsebusch et al. (21) did not find any evidence that participants with BII fear were more disgust sensitive than control participants, nor did they find any evidence that disgust elicits parasympathetic activation [see (22) for similar results; see also (23), for a recent meta-analysis on disgust proneness in other anxiety disorders].

### The Current Study

The current review focuses on well-known biases that reflect distorted processing in health as well as in psychopathology: (1) expectancy bias, in which individuals overestimate the likelihood of encountering the fear-relevant stimulus (encounter bias) or the negative outcome that will follow the encounter [consequence bias; for reviews on negative and positive expectancy bias, see (24, 25), respectively]; (2) attention bias, which is exhibited through faster engagement with and slower disengagement from the fear-relevant stimulus, followed by attentional avoidance of said stimuli [for reviews on negative and positive attention bias, see (26, 27), respectively]; (3) memory bias, in which individuals remember fear-related items more often than fear-unrelated items [for reviews on negative and positive memory bias, see (28, 29), respectively]; (4) perception bias, in which individuals overestimate a physical characteristic of the fear-relevant stimulus, such as its size or distance [e.g., (30–32); for a recent review, see (33)]; (5) interpretation bias, in which individuals interpret ambiguous stimuli as threatening [for reviews on negative and positive interpretation biases, see (34, 35), respectively]. Although these biases exist in healthy populations, they are more severe and persistent in populations with psychopathologies [for a review, see (36)].

Of note, each study that is presented in this review focused on one bias, although recently, efforts have been made to study biases in unison [for a recent review on the combined cognitive biases hypothesis, see (37)]. Thus, while the current review emphasizes the need to study cognitive biases in BII phobia, it also addresses the need to study the interaction between cognitive biases. Such an investigation could provide a more complete picture of cognition and cognitive patterns in BII phobia. Along the same lines, it is also important to study the interaction between cognition and emotion in BII phobia. Such an examination could help us understand whether BII phobia is unique in its cognitive patterns compared to other phobias, or whether it can be treated similarly to other disorders. Better understanding the cognitive mechanisms of BII phobia, especially in relation to other phobias and disorders, could help reach an answer.

The aim of the present article is to outline and integrate key findings on cognitive biases in BII phobia, to present existing treatment possibilities, and to suggest future research that may shed light on the specific characteristics of BII phobia and complement existing findings. Such research has important theoretical and clinical implications, as it can clarify whether BII phobia is indeed distinct from other phobias to develop more effective specific treatments, with specific emphasis on cognitive therapy.

## Methods

### Identification of Relevant Studies

Studies were identified from the following electronic databases based on key search terms for all available years: PubMed, PsycINFO, and Web of Science. The search strategy included the term “blood injection injury phobia” in conjunction with each of the following search terms: attention bias, expectancy bias, covariation bias, memory bias, interpretation bias, perception bias, and cognitive bias. The reference lists of the included studies were also searched to identify further studies. The final database search was completed on 2 November 2020.

### Inclusion and Exclusion Criteria

Studies were included if they met the following criteria: (1) included at least two groups of participants—participants with clinical or subclinical BII and healthy control participants; (2) adult population; (3) experimental studies; (4) examined at least one cognitive bias; and (5) appeared in English-language peer-reviewed journals. Studies were excluded if they were purely theoretical (e.g., reviews), books, theses, or written in a language other than English.

### Selection of Studies

After removing duplicates, the first author (EA) reviewed the titles and abstracts of all the studies. If a decision to include an article could not be reached based on the title and abstract review, the full text was reviewed.

### Data Extraction

For each study included in the review, the following information was extracted: experimental setup, participant characteristics, stimuli used, and main findings (see Tables 1–**3**).

**Table 1:** Summary of studies on attention bias in BII phobia.

**Study**	**Experimental setup**	**Participants**	**Stimuli**	**Main findings**
Armstrong et al. (38)	Eye movements—passive view	Participants with high injection fear (*n* = 33), participants with low injection fear (*n* = 32).	Pictures of the following categories: injection (needle puncturing skin), attack (aggressive dogs), appetitive (desserts), neutral (household objects).	• Engagement: high fear participants engaged with all emotional pictures more often than with neutral pictures (i.e., general hypervigilance). • Disengagement and avoidance: high fear participants rapidly disengaged from and spent less times viewing injection images, compared with low fear participants. • Attentional avoidance uniquely predicted behavioral avoidance from a syringe.
Buodo et al. (39)	ERP (P300 and slow waves)—free viewing	Participants with blood phobia (*n* = 23), participants with no phobia (*n* = 26).	Pictures of the following categories: pleasant (erotic couples and sport/adventure); neutral (household objects and neutral people); unpleasant (attacking humans—threat-irrelevant and mutilated bodies—threat relevant).	• No effect of picture category was found on free viewing time between groups. • A trend for visual avoidance of mutilations was found among participants with blood phobia.
Buodo et al. (40)	MEG (occipito-parietal activation 190–250)—passive viewing	Participants with high blood fear (*n* = 7), participants with no blood fear (*n* = 7).	Pictures of the following categories: pleasant (erotic couples, sports); aversive (threat, mutilations), neutral (household objects); minor injuries.	• High fearful participants exhibited stronger activity patterns for all pictures, compared with the no fear group (i.e., general hypervigilance). • Activity patterns did not differ between groups when blood-related stimuli were presented.
Buodo et al. (41)	ERP (N2pc)—detection of luminance changes of fixation with bilateral paired pictures	Participants with BII phobia (*n* = 12), participants with no phobia (number of participants is not mentioned).	Pictures of the following categories: injury (small injuries and minor surgical procedures—disorder-relevant); attack (attacking humans and aimed weapons—non-specific unpleasant); neutral (household objects, neutral people, and landscapes).	• When paired with neutral stimuli, injury pictures elicited larger early N2pc amplitudes than attack pictures in participants with BII phobia compared to control participants.
Elsesser et al. (42)	Behavioral - dot-probe and Stroop; HR was measured during passive view	Participants with various small animals phobias (*n* = 23), natural environment phobias (*n* = 5), situational phobias (*n* = 10), blood-injury phobia (*n* = 6), healthy control participants (*n* = 39).	Pictures of real, phobia-relevant situations (e.g., participants with height phobia were presented pictures of narrow passages and lifts).	• Participants with specific phobias exhibited accelerated HR when presented with phobia-relevant pictures, compared with control participants. This effect significantly correlated with the Stroop interference effect. • Behaviorally, no differences in attentional bias were found between groups on the dot-probe task. • Early deceleration of the HR reaction toward phobia-relevant pictures correlated with more pronounced selective attention (in the dot-probe task) toward these pictures among participants with phobia.
Haberkamp and Schmidt (43)	Behavioral – response priming paradigm	BII fearful (*n* = 19) and non-anxious control participants (*n* = 23).	Pictures of minor injury and non-injury (corresponding unharmed body parts).	• Phobia-relevant pictures lead to larger priming effects and faster reaction times in participants with BII fear, compared with neutral pictures. This effect was not found in control participants.
Leutgeb et al. (44)	ERP (P100, N100, P200, N200, P300, LPP) -discrimination task: real blood or not.	Participants with BII phobia (*n* = 20), participants with no phobia (*n* = 20).	Pictures of pig blood, water with red food coloring, water with pink food coloring.	• P100: In response to blood picture, participants with BII phobia showed higher amplitudes compared to control participants. Additionally, in the phobia group, amplitudes were higher toward blood pictures compared with pink fluid pictures. This effect was not found in the control group. • P300: Regardless of the fear group, participants exhibited larger amplitudes to blood pictures compared to other fluids. Classification accuracy did not differ between the fear groups.
Mogg et al. (45)	Behavioral – dot probe	Participants with high trait anxiety (*n* = 15), low trait anxiety (*n* = 15), high blood-injury fear (*n* = 11), medium blood-injury fear (*n* = 16), and low blood-injury fear (*n* = 13).	Pictures of high threat and mild threat (mutilation, injury, death, violence, warfare, and aggressive animals) and non-threat pictures.	• Participants with high levels of trait anxiety exhibited initial vigilance toward threatening stimuli, more so than participants with low levels of trait anxiety. However, the same participants did not exhibit subsequent avoidance of said stimuli. • Participants with high levels of BI fear exhibited initial vigilance in addition to subsequent avoidance of threatening stimuli, compared with neutral stimuli. Fear groups differed in avoidance, but not in vigilance.
Sarlo et al. (46)	ERP (N100, P200, P300, LPP) – passive view and repetitive presentation	Participants with BII phobia (*n* = 13), healthy control participants (*n* = 12).	Pictures of blood, mutilations, and neutral pictures.	• ERP: Participants with BII phobia exhibited larger N100 and smaller LPP amplitudes toward mutilation pictures, compared with control participants. • Subjective ratings: participants with BII phobia indicated a linear increase of subjective arousal over time (i.e., sensitization), while control participants did not.
Sawchuk et al. (9)	Behavioral – Stroop	Participants with BII phobia (*n* = 53)^*^, participants without phobia (*n* = 54)	(1) Films for mood induction: disgust (scenes of insect maggots and larvae) and neutral (aerial landscape scenes). (2) Stem completion task, primed using a Stroop task. Word categories: medical, disgust-related, negative and neutral.	• No attention bias for medical and disgust-related words was found, even when disgust was induced using films.
Wenzel and Holt (47)	Behavioral – dot probe	Participants with BII phobia (*n* = 14), spider phobia (*n* = 13), and non-anxious control participants (*n* = 14).	Words: spider-related, blood-related, positive, negative, and neutral.	• No attention bias toward phobia-relevant words was found.

* *These participants should be considered subclinical, as they were not formally diagnosed. Rather, they were divided into groups based on relevant questionnaires. Due to the fact that individuals with BII phobia mostly report feelings of disgust, the authors found it inaccurate to refer to them as “fearful” participants and so they were referred to as “phobic” participants*.

## Results

**Figure 1:**
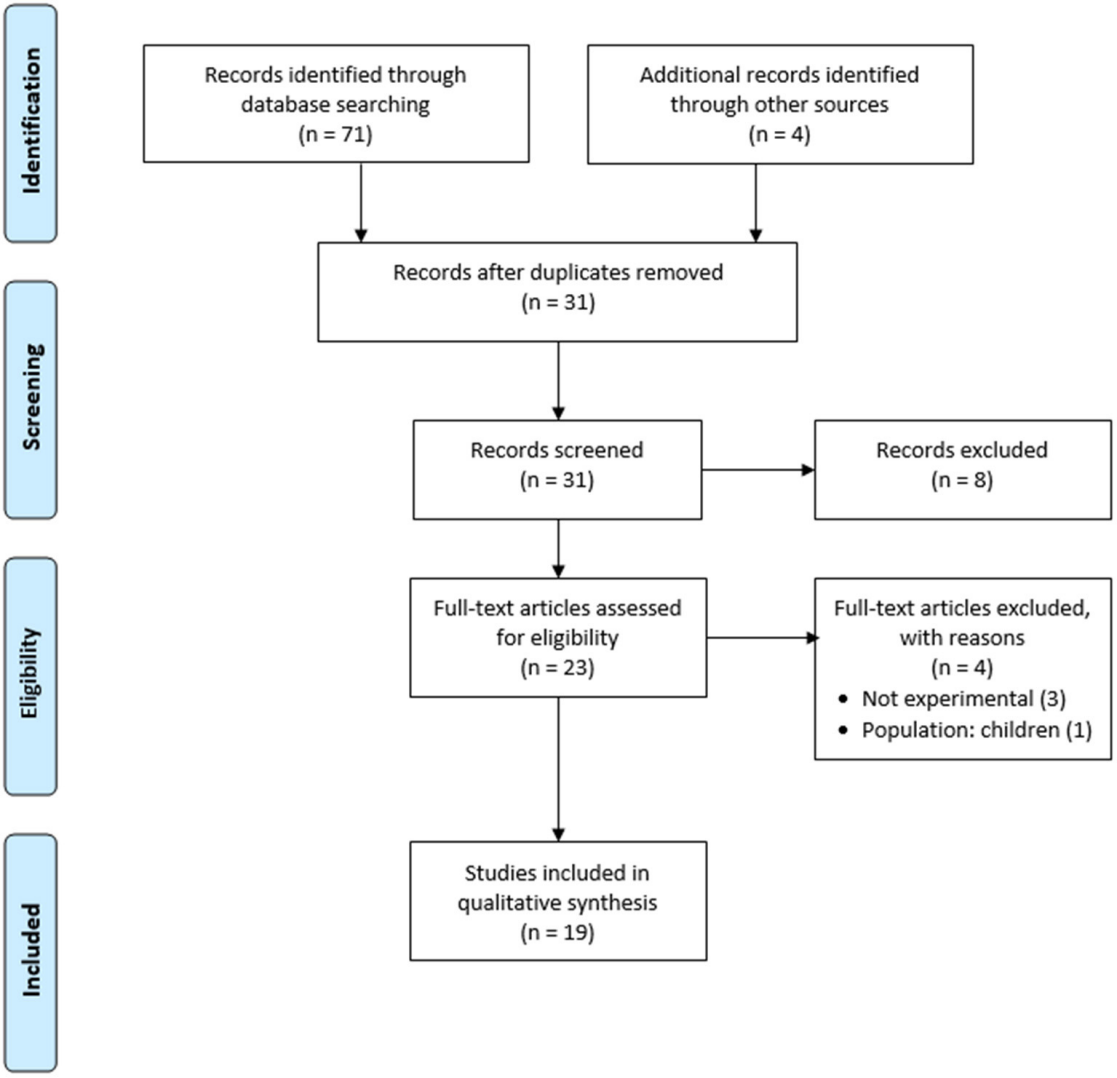
Flow chart of data selection for the review.

The electronic search retrieved 71 studies. An additional four studies were added manually from hand searching the reference lists of the retrieved studies. After duplicates were removed, a total of 31 studies remained. A review of the titles and abstracts resulted in excluding eight studies that did not meet the eligibility criteria. After exclusion based on the titles and abstracts, 23 articles were retained for full-text review. Following the full-text review, four articles were excluded from the review for the reasons highlighted in the flow chart shown in Figure 1. A total of 19 studies met the inclusion criteria and were included in the review. One of the articles examined two biases simultaneously: attention and memory biases. The included studies encompassed a total of 1,201 participants: 506 participants with BII phobia/high BII fear levels, 538 healthy control participants, and 157 participants with other specific phobias or anxiety disorders. The search yielded findings on only three biases: attention bias, expectancy bias, and memory bias. No studies were found on perception bias or interpretation bias.

### Attention in BII Phobia

Attention bias plays a crucial role in the maintenance and etiology of anxiety disorders [for a review, see (26). Attention bias includes three main components: faster engagement with and slower disengagement from threatening stimuli compared to neutral stimuli, followed by attentional avoidance of the same stimuli. Thus, attention bias is characterized by a vigilance-avoidance pattern [for a review, see (56); for a meta-analysis, see (57)]. Of note, not all studies support the vigilance-avoidance model. For instance, some studies suggest that difficulty in disengagement and avoidance occur simultaneously, with disengagement occurring covertly and avoidance occurring overtly (58). Additionally, attention bias is exhibited differently in various disorders and using various tasks [e.g., anxiety vs. depression; for a review, see (59).

Our literature search yielded 11 studies relevant to attention bias in BII phobia (see Table 1 for a detailed summary of each study). Together, these studies include 548 participants: 211 participants with BII phobia/high BII fear levels, 240 control participants and 97 participants with other specific phobias or anxiety disorders.

Sawchuk et al. (9) failed to find attention bias toward medical and disgust words using a Stroop task in participants with and without BII phobia. Similarly, Buodo et al. (39) attempted to find attention bias in BII phobia using event-related potential (ERP) methods. Participants with BII phobia and control participants looked at threat-relevant, emotional threat-irrelevant and neutral pictures. For both groups, late attentional components (P300 and slow waves) did not differ between the different picture categories. Nevertheless, a trend toward visual avoidance (measured as free viewing time) of the threat-relevant pictures was found in the phobia group.

Buodo et al. (40) also attempted to find attention bias using magnetoencephalography (MEG) while presenting blood-related and unrelated pictures to high BII fearful and non-fearful participants. Compared to the non-fear group, the BII fear group exhibited enhanced activity at occipito-parietal sites between 190 and 250 ms after picture onset for aversive, pleasant and neutral pictures. These results suggest the existence of general hypervigilance in participants with BII phobia, regardless of stimuli valence. Moreover, this activity did not differ between the fear groups when viewing blood-related stimuli, suggesting that BII-related stimuli are generally aversive, regardless of fear levels.

Furthermore, Leutgeb et al. (44) showed pictures of blood and of water with red or pink food coloring to participants with and without BII phobia. Results showed early selective attention (P100 amplitudes: 90–140 ms) toward pictures of blood compared to pictures of pink or red colored water among participants with BII phobia, while later components (P300: 340–500 ms) revealed that both groups of participants exhibited selective attention toward blood compared to pink or red colored water. These results suggest that participants with BII phobia exhibit attention bias during early processing, while all participants exhibit attention bias toward blood during later stages of processing, reflecting a general human tendency to exhibit attention bias toward blood-related stimuli.

Buodo et al. (41) demonstrated attention bias in participants with BII phobia using N2pc measurements in a visual detection task. Participants with and without BII phobia were shown injury, attack, and neutral pictures, with one of the unpleasant pictures always presented simultaneously with a neutral picture. Injury pictures elicited larger N2pc activation than attack pictures when paired with neutral pictures only in the group with BII phobia. Only early N2pc (180–240 ms post-stimulus) was enhanced, while late N2pc (240–310 ms post-stimulus) did not differ as a function of picture type. Thus, similar to Leutgeb et al. (44), these results suggest that attention bias in BII phobia can be detected beginning in early processing stages [see also (43), for a behavioral study that found attention bias in BII during early processing stages]. Additionally, similar to Buodo et al. (39), these results suggest later attentional avoidance among participants with BII phobia. Taken together, the aforementioned studies tentatively suggest the existence of a vigilance-avoidance pattern in individuals with BII phobia.

Along the same lines, using repetitive presentation of different BII-related pictures, Sarlo et al. (46) found attention bias in BII phobia during early and late processing stages. Pictures of blood and mutilations were randomly presented while interspersed with neutral pictures. This continuous presentation of fear-related stimuli led to cognitive-emotional sensitization in the phobia group, but not in the non-phobic group. Hence, participants with BII phobia exhibited a linear increase in subjective arousal over time for all picture categories. In addition, similar to Buodo et al. (41), a vigilance-avoidance pattern was found in the BII phobia group, as during early processing stages participants with BII phobia exhibited selective processing of BII-related pictures, while during later stages they exhibited cognitive avoidance of these stimuli.

Using eye movement measurements, Armstrong et al. (38) provided partial support for the existence of attention bias in BII fear. While highly fearful BII participants attended to BII-related pictures more often than those who had low levels of fear, these highly fearful participants did not attend to BII-related pictures more often than to other emotional pictures. Attention was calculated as the number of trials in which a specific type of picture captured the initial fixation. In addition, a strong avoidance effect was found, as highly fearful BII participants quickly disengaged from BII-related pictures compared to other pictures, while this difference was not found in the low BII fearful group. The finding regarding vigilance supports previously mentioned studies [e.g., (40)] in which general hypervigilance was found in BII fearful participants.

Studies examining attention bias in BII phobia rarely use classic attention bias paradigms [for reviews, see (26, 60)], making it harder to compare attention bias in BII phobia with other phobias. Although such paradigms may be inconsistent as well in that they do not always reveal attention bias in anxious participants (61), they can still offer a solid beginning for research in this area. These paradigms can be finely tuned once a solid base of existing research regarding BII phobia is established. In the study of BII, attention bias is often assessed using other measures, most of which are physiological. Thus, these measures, which are often more implicit and more difficult to interpret than traditional behavioral paradigms that measure attention, may be less sensitive in the detection of attention bias in BII phobia [for an elaboration on the advantages of behavioral research, see (62)].

There are, however, a few exceptions: Wenzel and Holt (47) used the classic dot-probe paradigm to compare BII phobia with spider phobia. Blood-related, spider-related, positive, negative and neutral words were presented. Attention bias was not found in any fear group, leading the researchers to conclude that semantic stimuli may not be the most effective stimuli for the detection of attention bias. Similarly, Sawchuk et al. (9) did not find a Stroop interference effect toward BII-related words among participants with BII phobia. On the other hand, Elsesser et al. (42) found increased HR toward phobia-related stimuli and a significant Stroop effect in various phobia groups (i.e., participants with BII and with small animal, environmental, and situational phobias). Nevertheless, Elsesser et al. (42) did not find a biphasic response in the BII phobia group, and no significant dot-probe effect emerged in any of the phobia groups. Lastly, Mogg et al. (45) used the dot-probe paradigm to examine differences in the vigilance-avoidance pattern in participants with trait anxiety and with BII fear, using different exposure durations (500 and 1,500 ms). Results showed that while participants with trait anxiety exhibited vigilance without avoidance, participants with BII fear exhibited both initial vigilance and later avoidance (45). These results further suggest that attention bias may vary between disorders and between different paradigms.

To summarize, the literature provides mixed results regarding the existence of attention bias in BII fear and phobia. While some studies suggest general hypervigilance toward emotional stimuli among participants with BII regardless of the relatedness of these stimuli to BII, other studies found domain-specific vigilance-avoidance patterns toward BII-related stimuli, and still other studies only found disorder-specific avoidance without preceding vigilance effects. In addition, sometimes the bias was found within-participants (i.e., toward BII-related stimuli compared with neutral stimuli), while at other times it was found between-participants (i.e., between participants whose fear of BII-related stimuli was low and high). One reason for these inconsistencies may be that unlike fear, disgust has been suggested to lead to avoidance for evolutionary reasons, as it diverts attention away from the relevant stimulus rather than maintaining it (63). This difference was greater among participants with high disgust sensitivity (63). Thus, disgust and individual disgust sensitivity may affect attentional allocation.

Another reason for the mixed findings may be related to the required performance, namely to whether the task involved active responding or passive viewing. The tasks also varied in the stimuli presented and in the balance between positive, negative, and phobia-related stimuli, which can affect expectancies and in turn, performance. Additionally, varying paradigms, timings, tasks and methodologies were used in the reviewed studies, also potentially contributing to the reported inconsistencies.

### Expectancy in BII Phobia

Expectancy can be defined and measured in several ways. First, a-priori expectancy is present prior to stimulus presentation. There are two types of biased a-priori expectancy (64): *encounter bias*, in which individuals overestimate the likelihood of encountering the fear-evoking/threatening stimulus; and *consequence bias*, in which individuals overestimate the extent of the negative consequence following the encounter with the fear-evoking/threatening stimulus. Second, a-posteriori bias occurs after stimulus presentation. In this bias, individuals overestimate having encountered the stimulus or its consequences (similar to a-priori encounter and consequence biases, respectively). As this bias is measured after the threat-relevant stimulus has been presented, it is also considered a memory bias [for a review, see (36)].

Expectancy and expectancy bias have been studied to some extent in the context of anxiety disorders and specific phobias [for a review, see (25)], respectively], but less so in BII phobia. Nonetheless, several studies have examined the role of expectancy in pain and hospital settings (65). While pain expectancy can be adaptive in that it prepares us for certain threatening situations (66), expectancy bias in BII phobia can serve to maintain the avoidance of important medical procedures.

Our literature search yielded six relevant studies about expectancy/covariation bias in BII phobia (see Table 2 for a detailed summary of each study). Together, these studies include 392 participants: 195 participants with BII phobia/high BII fear levels and 197 control participants.

**Table 2:** Summary of studies on expectancy bias in BII phobia.

**Study**	**Experimental setup**	**Participants**	**Stimuli**	**Main findings**
Connolly et al. (48)	Behavioral – illusory correlation paradigm	Participants with high BII fear (*n* = 32) and low BII fear (*n* = 30).	Pictures of: disgust (vomit and human feces); fear (vicious dog and a man with a knife); and neutral (flowers and a chair). Outcome stimuli consisted of human facial expressions portraying each emotion.	• Greater covariation bias for generally affective stimuli was found in high fear participants compared to low fear participants.
de Jong and Peters (49)	Behavioral – illusory correlation paradigm	Participants with high BII fear (*n* = 25) and low BII fear (*n* = 27).	Fear-relevant pictures: blood-donation; fear-irrelevant pictures: rabbit, flower. Outcome stimuli: electrical shock (harm), drinking a harmless but bad tasting fluid (disgust) or no outcome (neutral).	• Both groups of participants exhibited a-priori expectancy bias (blood-related slides and disgust- or harm-relevant outcomes). • Neither fear group exhibited covariation bias.
Olatunji et al. (50)	Behavioral – evaluative learning	Exp. 1: 60 unselected participants; Exp. 2: participants with BII phobia^*^ (*n* = 13), control participants with no phobia (*n* = 11).	Exp. 1: CS: neutral facial expressions; UCS: fear (snakes, spiders); disgust (e.g., rotting foods, body products); and neutral (e.g., tools, appliances). Exp. 2: same CS as Exp. 1; UCS - blood, injections, bodily injury.	• Exp. 1: No differences between conditions were found. Within-subject comparisons showed that post-experimental ratings of fear and disgust were higher among expressions that were paired with any type of pictorial stimuli, compared to unpaired expressions. • Exp. 2: Post-experimentally, both fear groups rated faces that were paired with BII-related stimuli as more disgusting but not more fearful, compared to pre-experimental ratings.
Pury and Mineka (51)	Behavioral – illusory correlation paradigm	Exp. 1: participants with high blood-injury fear (*n* = 18), participants with low blood-injury fear (*n* = 22); Exp. 2: participants with high blood-injury fear (*n* = 15), participants with low blood-injury fear (*n* = 25); Exp. 3: participants with high blood-injury fear (*n* = 17), participants with low blood-injury fear (*n* = 12).	Fear relevant pictures: mutilation, surgery, minor injuries. Fear irrelevant pictures: babies, flowers, rabbits. Outcome: shock (aversive), tone or no outcome (neutral outcomes).	• In all experiments and all fear groups, participants exhibited covariation bias toward blood-injury-related stimuli, compared to other stimuli/combinations.
Van Overveld et al. (52)	Behavioral – thought experiment	Participants with high levels of blood fear (*n* = 30) and low levels of blood fear (*n* = 30).	Slides of the following categories: blood (small bloody wound on someone's leg), fear (gun pointed at the viewer), disgust (maggots), fear and disgust (growling dog), and neutral (rabbit). Possible outcomes: electrical shock, drinking a nauseating juice or no outcome.	• Both groups of participants exhibited a-priori expectancy bias (expected shock and juice to follow blood). This effect was stronger in participants with high fear levels, compared to low fear levels.
Wenzel and Golden (53)	Behavioral – listing events	Participants with blood fear (*n* = 45), participants with no fear (*n* = 40).	Scenarios about: blood, injections, hospitals, or injury.	• After reading the scenarios, participants with blood fear listed more negative emotional and physiological events/experiences than participants with no fear.

* *These participants should be considered subclinical, as they were not formally diagnosed. Rather, they were divided into groups based on relevant questionnaires. Due to the fact that individuals with BII phobia mostly report feelings of disgust, the authors found it inaccurate to refer to them as “fearful” participants and so they were referred to as “phobic” participants*.

For the most part, expectancy studies have used a covariation paradigm to examine whether participants with BII phobia exhibit a-posteriori bias toward BII-related stimuli. In this paradigm, several stimuli (e.g., negative and neutral) are typically randomly paired with several outcomes (e.g., aversive and neutral, such as electrical shock or no outcome). Post-experimentally, anxious participants tend to overestimate the pairing of aversive stimuli with negative outcomes compared to neutral outcomes. Pury and Mineka (51) randomly paired aversive or neutral outcomes (shock, neutral tone, or no outcome) with BII-related and neutral pictures presented to participants with low and high BII fear levels. Results showed that post-experimentally, both fear groups equally overestimated the co-occurrence of BII-related pictures with a shock, indicating the existence of a-posteriori/memory bias in all participants, regardless of their pre-existing fear levels.

Using the same paradigm, de Jong and Peters (49) found somewhat different results. They presented neutral and BII-related pictures with different outcomes: no consequences (neutral outcome), drinking 5 ml of harmless but bad tasting solution (disgusting outcome), or receiving an electrical shock (harm-related outcome). Participants had low and high BII fear levels. A-priori consequence bias was found, such that all participants overestimated the likelihood of co-occurrence between BII-related pictures and disgust- and harm-related outcomes [see (52), for similar results using a thought experiment; of note, in (52), the bias was stronger in the high BII fear group, but still present in the low fear group]. Nevertheless, all participants went through online adjustment as a-posteriori expectancy bias was not found (i.e., they managed to change their expectation during the experiment between the a-priori and a-posteriori measurements). Thus, both fear groups underwent a learning process in which they (likely unconsciously) realized that the stimuli were randomly paired with the outcomes.

These varying outcomes may possibly be explained by the use of different pictures. De Jong and Peters used pictures “in a positive context” that included blood donations, while Pury and Mineka used explicitly aversive pictures, ranging from minor injuries to mutilations. Another source of variance is the inclusion of a “disgust-related outcome” condition [present in (49), but absent in Pury and Mineka (51)], which may act as an alternative to the harm-related outcome condition, thus presenting two possible negative outcomes instead of just one.

Along similar lines, Olatunji et al. [(50); Exp. 2] paired neutral facial expressions with BII-related pictures. Post-experimentally, both low and high BII fear groups evaluated the neutral expression as significantly more disgusting than their pre-experimental evaluation. A similar non-significant trend was found in participants' evaluation of fear (i.e., post-experimentally, both groups rated the neutral expression as more fear-evoking than their pre-experimental evaluation). The authors concluded that these results reflect the fact that BII-related stimuli are associated a-priori with disgust and that fear is a secondary emotion for these stimuli. Thus, the a-priori expectancy or readiness to associate BII-related stimuli with feelings of disgust and fear may facilitate the conditioning processes found in both studies.

Other studies using the same covariation paradigm opted to avoid using BII-related pictures entirely. In a study conducted by Connolly et al. (48), low and high BII fearful participants were shown disgusting (vomit and human feces), fear-evoking (vicious dog and man with a knife), and neutral (flowers and a chair) pictures. These were randomly paired with disgusted, fearful, and neutral facial expressions. These modifications were aimed at making the paradigm more ecologically valid. Unlike in other studies, in these studies a difference was found between low and high BII fearful participants, such that only high BII fearful participants exhibited a-posteriori bias (i.e., overestimated the co-occurrence between emotionally congruent stimulus and outcome). These results suggest that BII fearful participants may also be sensitive to stimuli that are generally disgusting and fear-evoking, even if these are not fear specific. Additionally, these findings reflect the importance of ecological validity when attempting to find differences between groups. Previous studies (51) may have found a ceiling effect due to the use of extremely aversive stimuli. Indeed, Pury and Mineka (51) found that both fear groups rated pictures of surgery and mutilations as highly unpleasant. While de Jong and Peters (49) tried to show pictures in a more positive context, they nonetheless presumably showed pictures of needles puncturing arms. Such situations may be considered aversive by both fear groups.

Consequence bias can also be measured using different paradigms. Wenzel and Golden (53) showed various scenarios concerning blood, injections, hospitals or injury to low and high fear participants. Participants were asked to list events and experiences that occur during such situations. The results showed that high BII fear participants listed more negative emotional and psychological events than low fear participants. The authors concluded that among participants with high BII fear, self-schemata for blood-related situations involve expectations of harm or threat. Additionally, the authors suggested that these results may indicate that participants with high BII fear interpret scripted information in an excessively negative manner, possibly suggesting the existence of an interpretation bias.

To summarize, the aforementioned studies suggest the existence of a-priori consequence bias toward BII-related stimuli. While some studies found a-posteriori expectancy bias, other studies found that the a-priori bias can be adjusted to eliminate a-posteriori bias. Note that most studies did not find a difference in expectancy bias between high and low fear groups, suggesting a general pre-disposition to fear and being disgusted by BII-related stimuli or a ceiling effect due to the highly aversive and arousing pictures used in these studies. These finding have important theoretical and clinical implications, as will be discussed in the discussion section.

### Memory Bias in BII Phobia

Memory bias refers to selective memory regarding threatening stimuli. Specifically, participants with a specific phobia or an anxiety disorder are likely to remember having faced threat-relevant stimuli more so than neutral stimuli [for a review on memory bias, see (28)]. Of note, a-posteriori expectancy bias may also be considered a memory bias, as it also reflects selective memory of past events.

Our literature search yielded three relevant studies about memory in BII phobia (see Table 3 for a detailed summary of each study). One of these studies was already mentioned above, as it also covers attention bias in BII phobia (9). Together, these studies include 368 participants: 153 participants with BII phobia/high BII fear levels, 155 control participants and 60 participants with other specific phobias or anxiety disorders.

**Table 3:** Summary of studies on memory bias in BII phobia.

**Study**	**Experimental setup**	**Participants**	**Stimuli**	**Main findings**
Sawchuk et al. (9)	Behavioral – implicit memory task (word stem completion)	Participants with BII phobia (*n* = 53)^*^, participants without phobia (*n* = 54)	Stem completion task, primed using a Stroop task. Word categories: medical, disgust-related, negative, and neutral.	• On the word stem completion task, participants with BII phobia completed more medical and disgust-related word stems, compared with control participants.
Sawchuk et al. (54)	Behavioral – recognition memory	Exp. 1: participants with BII phobia (*n* = 37)^*^, participants with spider phobia (*n* = 39), participants with no phobia (*n* = 40).Exp. 2: participants with BII phobia (*n* = 36)^*^, participants with no phobia (*n* = 36).	Pictures of surgical operations (phobia relevant), spiders (phobia relevant), rotting food (general disgust), body products (general disgust), flowers (neutral).	• Exp. 1: No differences emerged between participants with BII phobia, spider phobia or control in discrimination ability or response bias for any picture category. Results showed that all participants responded in a liberal manner toward and surgical and disgust pictures, and in a conservative manner toward spiders pictures. • Exp. 2: Once again, no differences between groups emerged in discrimination ability or response bias for any picture category, either at 50 or 500 ms exposure durations. Again, in the 500 ms exposure duration condition, participants responded in a liberal manner toward surgical and disgust pictures.
Wenzel et al. (55)	Behavioral – memory retrieval after cue presentation	Participants with blood-injury fear (*n* = 27), participants with spider fear (*n* = 21), participants with no fear (*n* = 25).	Cue words: blood/injury-related, spider-related, neutral.	• Participants in both fear groups retrieved more negative memories compared to control participants, despite not having reported higher levels of anxious or depressive symptoms.

* *These participants should be considered subclinical, as they were not formally diagnosed. Rather, they were divided into groups based on relevant questionnaires. Due to the fact that individuals with BII phobia mostly report feelings of disgust, the authors found it inaccurate to refer to them as “fearful” participants and so they were referred to as “phobic” participants*.

As mentioned above, in a Stroop task Sawchuk et al. (9) failed to find attention bias toward medical and disgust words in participants with and without BII phobia. These researchers did, however, find, another cognitive bias: memory bias. An implicit memory task showed that participants with BII phobia completed more BII-related words than participants with no phobia. Later, Sawchuk et al. (54) used analysis of decision-making criteria to attempt to detect memory bias toward fear-relevant stimuli in participants with small animal phobia and BII phobia. To this end, they measured discrimination ability (*d*′) and response bias (*c*′). Results showed that all participants (participants with spider phobia, BII phobia and no phobia) adopted a liberal criterion toward surgical and disgust pictures and a conservative criterion toward spider pictures. Thus, all participants exhibited a general memory bias toward surgical and disgust pictures, while all participants exhibited a lack of bias toward spiders. Once again, this may be due to a ceiling effect, as extremely aversive surgical and disgusting pictures were shown (surgical operations, body products, and rotting foods), with only one neutral category (i.e., flowers). The authors claim that one reason for these results may be that evaluations are primarily based on disgust rather than fear, and thus a bias is only exhibited toward the more disgusting stimuli. This claim is based on participants' subjective ratings of the pictures, as all participants rated spiders as more fearful than disgusting, while the opposite pattern was found for surgical and disgusting pictures.

Another study used memory retrieval to determine whether BII phobia is characterized by a memory bias. Specifically, participants with spider fear, blood/injury fear, and no fear were presented with spider-related, blood/injury-related, and neutral cue words. Participants were asked to retrieve the first specific personal memory that came to their minds. Results showed that both fear groups retrieved a higher percentage of negative memories than non-fearful participants, despite the fact that neither fear group reported higher levels of anxious or depressive symptoms (55).

To summarize, studies on memory bias in BII phobia show mixed results. This may be due to the fact that only three relevant studies were found, and each study used a different method to assess memory bias. Future studies should conduct more thorough and systematic examinations of memory processes in BII phobia.

### Interpretation Bias in BII Phobia

Our search did not yield any relevant results for interpretation bias in BII phobia.

### Perception Bias in BII Phobia

Our search did not yield any relevant results for perception bias in BII phobia.

## Discussion

The aforementioned studies suggest that attention, expectancy and memory biases are involved in BII phobia. Nevertheless, the extent of the involvement of each bias in the disorder remains unknown, especially when taking into consideration how the same biases are better understood in other specific phobias and anxiety disorders. In addition, the exact differences between the disorders are unclear. Similarly, both fear and disgust seem to play a part in BII phobia and in other specific phobias, though to different extents. Perhaps these differences in emotions also lead to differences in distorted cognitive processing.

These findings lead to the conclusion that there is room for more cognitive research about BII phobia. While this phobia seems to be unique, the results are inconsistent and most of the existing studies are not recent. One reason for these inconsistencies may be the fact that some studies used highly aversive, arousing, and negative images, which may be considered extremely unpleasant even by participants with no phobia. Moreover, some studies used several BII-related, disgust-related, and/or generally aversive/unpleasant categories, with only one neutral category, thus making the pictures imbalanced and setting a generally negative context for the experiment. Additionally, the reviewed studies employed highly varied methods, also potentially explaining some of the inconsistencies.

Future studies should consider additional cognitive aspects, such as sense of control or perceived control, which may mediate fear of BII-related stimuli (7). Similarly, intolerance of uncertainty plays a crucial role in all anxiety disorders, but it has yet to be extensively studied in specific phobias [for a review, see (67)]. Along these lines, while not many studies have focused on attention and expectancy biases, even fewer have examined other biases such as memory bias in BII phobia and fear. Additionally, no relevant research was found on interpretation and perception biases.

Oar et al. (68) suggested an integrative developmental model of BII phobia. The model includes biological vulnerability factors, such as genetics, neurobiology, evolutionary preparedness, predisposition to fainting, and pain sensitivity. These factors interact with temperament (behavioral inhibition, disgust sensitivity) and social learning influences throughout development (e.g., direct/vicarious conditioning, negative information transmission). All the aforementioned factors then affect hypervigilance and selective attention to threat, triggers (i.e., BII-related stimuli and physical symptoms), and dysfunctional information processing and phobic beliefs (i.e., attentional biases, overestimation of threat, underestimation of coping, appraisals of disgust, and perceived control). In turn, these factors all affect BII phobia, which is maintained by behavior (i.e., avoidance and safety behaviors) as well as by physiological and affective experiences (i.e., fear, disgust, pain, faintness, nausea, and embarrassment), which then reinforce selective attention to threat, triggers, and dysfunctional information processing. Later on, Oar et al. (69) also showed that a-priori encounter and consequence biases differ between BII phobia and dog phobia.

The current review further supports including biased expectancies and attention in BII phobia, as well as contrasting BII phobia with other specific phobias, thus suggesting that BII phobia is indeed unique.

Recent research efforts have focused on studying the interaction between cognitive biases rather than on each one in isolation [for a recent review on the combined cognitive biases hypothesis, see (37)]. According to this approach, studying the interaction between biases is more fruitful and more ecological, as biases seldom exist in isolation (70). Correspondingly, a series of studies addressed the causal interaction between expectancy and attention bias in spider phobia (71–73). Moreover, manipulation of expectancies and frequencies can reduce attention bias in fear of spiders (74). Thus, integrating between cognitive biases is crucial for a deeper understanding of cognitive processes in anxiety and can help in developing effective cognitive bias modification methods (75) aimed at enabling concerned individuals to undergo potentially life-saving medical procedures.

In general, specific phobias and anxiety disorders are often treated effectively using cognitive behavioral therapy [CBT; for a recent meta-analysis, see (76)]. For instance, Lilliecreutz et al. (77) found that CBT effectively reduced BII symptoms in a group of pregnant women, compared to a group of pregnant women with untreated BII phobia and to a group of healthy pregnant women. The treatment included prolonged exposure to and education about the different functions of lancets, syringes, injection needles, and intravenous catheters, as well as exposure to various phobic situations, such as insertion of an intravenous catheter.

Another behavioral treatment found to be effective for BII phobia entails applied tension and *in-vivo* exposure [for a review, see (78)]. Applied tension is specifically tailored for participants with BII, due to the disorder's unique physiological (i.e., biphasic) response. Specifically, participants are taught a coping skill that reverses the second part of the biphasic response. Thus, participants learn to identify the earliest signs of drops in BP and to apply tension by tensing their gross body muscles, thus avoiding fainting (79). Of note, participants need to use the technique before encountering difficult situations (i.e., start when disinfectant is applied to their skin before a blood drawing procedure). Otherwise, it often is too late, and patients will faint, despite attempts to use applied tension. Treatment that combined CBT with applied tension was also found to be effective in a case study of a man with BII phobia [(80); see also (68, 81), for CBT in children and adolescents with BII phobia]. Of note, applied tension could also be considered a CBT technique. In reviewing the literature on treatments for BII phobia, Ayala et al. (82) found that applied tension has no added benefit beyond *in-vivo* exposure alone.

The evidence in the current article may help improve treatment of BII phobia by treating various cognitive biases. Note that cognitive biases can be considered determinants of vulnerability and resilience in anxiety disorders, thus making their identification particularly important in clinical settings [for a review, see (83); see also (84)]. For instance, reducing a-priori expectancies and irrational beliefs about what may happen when encountering a BII-related situation may prevent or reduce the first part of the biphasic response (i.e., increased HR and BP). Similarly, reducing attention bias has also proved to be an effective means of reducing fear in specific phobias and other anxiety disorders [for a review, see (61)]. Additionally, addressing feelings of disgust may also prove to be helpful, as disgust also plays a role in exposure therapy in BII phobia (85)]. Thus, cognitive and behavioral treatment can complement each other, as they aim at reducing the effects of the two phasic components associated with BII phobia. Note that despite the many recent and extensive studies, reviews and meta-analyses of treatments for phobia and anxiety disorders, studies focusing on treatments for BII phobia are quite infrequent and mostly older, despite their clinical significance.

Attention bias is believed to exert a causal influence on anxiety symptoms, and vice versa [for a review, see (61, 86); see also (87) for a critical review]. Thus, attention bias modification (ABM) paradigms aim at reducing attention bias, thereby reducing anxiety symptoms. In an extensive review of studies using different paradigms to measure attention bias across varying disorders conducted by Van Bockstaele et al. (61), BII phobia was never involved, while other specific phobias such as snake and spider phobias were mentioned often. Moreover, examination of attention bias in these disorders led to relatively consistent results across different paradigms. Hence, systematic examination of the existence of attention bias in BII phobia (or lack thereof) may be of crucial theoretical and clinical value. As mentioned earlier, expectancy has been found to have a causal impact on attention bias, and thus the study of both biases is of importance.

As mentioned earlier, reduction of attention bias can also be achieved using manipulations of expectancy and frequencies [e.g., (74)]. Expectancy manipulation can also be used with applied tension, to maximize the treatment's benefits. For instance, individuals' physical responses can be regarded as expectancy cues to prepare individuals for the upcoming exposure to BII-related stimuli and to help them apply tension in time. Along the same lines, applied tension could also be applied with various cognitive bias modification treatments. Emotion regulation strategies may also facilitate treatment in BII phobia (18).

To conclude, BII phobia has been understudied to a great extent, especially compared to the literature about other specific phobias. While some studies have examined the physiological aspects of BII phobia, research has for the most part overlooked its cognitive aspects. The review of expectancy and attention biases in BII phobia provided in this paper suggests that, as proposed by Sawchuk et al. (9), information processing in BII phobia is qualitatively different than in other specific phobias. Thus, a better understanding of the cognitive aspects of this disorder may lead to the development of more effective treatments.

## Author Contributions

EA conducted literature searches and provided summaries of previous research studies and wrote the first draft of the manuscript. HO-S and TA contributed and helped rewrite and revise subsequent versions. All authors contributed to and have approved the final manuscript.

## Conflict of Interest

The authors declare that the research was conducted in the absence of any commercial or financial relationships that could be construed as a potential conflict of interest.
